# Temper the Specialist Nurses Heterogeneity in the Interest of Quality Practice and Mobility—18 EU Countries Study

**DOI:** 10.3390/healthcare10030435

**Published:** 2022-02-25

**Authors:** Nico Decock, Adriano Friganovic, Biljana Kurtovic, Ber Oomen, Patrick Crombez, Christine Willems

**Affiliations:** 1 Nurse Anesthesia School, University Hospital of Lille, 59000 Lille, France; nico.decock@cnpia.fr; 2 Department of Anesthesiology, Reanimatology and Intensive Care, University Hospital Centre Zagreb, 10000 Zagreb, Croatia; adriano@hdmsarist.hr; 3 Department of Nursing, University of Applied Health Sciences, 10000 Zagreb, Croatia; 4 European Specialist Nurses Organization, 1000 Brussels, Belgium; ber.oomen@live.nl (B.O.); patrick.crombez@bordet.be (P.C.); 5 Department of Clinical Hematology and Cytapheresis, Jules Bordet Institute, 1000 Brussels, Belgium; 6 The Institut Supérieur d’Enseignement Infirmier, University College Leonard de Vinci, 1200 Brussels, Belgium; christine.willems@vinci.be

**Keywords:** specialist nurses, European Union, recognition, harmonization

## Abstract

Background: The position of the specialist nurse profession varies across the European Union. Action is required to address the challenges to promote mobility and the contribution of specialist nurses to quality of care. The purpose of the study is to identify the interfaces of the specialist nurse profession across the European Union. Methods: A mixed method study was conducted in October 2019 and total of 40 answers from 18 different European Union countries were selected using a purposive sampling method. Results: The participants had completed various Bologna degree cycles and 57.2% had followed a specific educational programme to become a specialist nurse. More professional autonomy was acquired by 81.9% participants. Conclusion: A striving for homogeneity in the interpretation of the specialist nurses role and competencies is needed to achieve better quality of care provision and facilitate their mobility around the European Union. The lack of recognition identified in this study should encourage nurse managers to consider specialist nurse roles with the aim of capitalizing on the advanced care and expertise that specialist nurses provide. These results are an opportunity to improve the specialist nurses profession with an ultimate impact on management practices of streamlined, cost-effective clinical services.

## 1. Introduction

The International Council of Nurses defined a specialist nurse (SN) as “A nurse prepared beyond the level of a general nurse and authorized to practice as a specialist with advanced expertise in a branch of the nursing field. Specialist practice includes clinical, teaching, administration, research and consultant roles” [1]. Since the beginning of the 21st century, the roles, regulation, and competencies of SNs have been extensively described [2,3,4,5,6], but their implementation within the European Union (EU) has been left to Member States’ own decisions. For example, some specialist education programmes are organized by universities, and some are under the jurisdiction of other educational or health institutions [7]. Following such examples in the past decade, there has been a further proliferation of the meaning of the SN title in Europe. There are a great variety of descriptions and understanding of SN roles [8,9,10], as well as diversity in the education, conditions, recognition, autonomy, expected responsibilities, and scope of work of SN nurses across the EU and even within the Member States themselves [5,11,12,13]. The term ‘Specialist Nurse’ as used by authors in this article also includes the varieties of the nurse profession at the specialist and/or higher education level such as ‘Advanced Practitioner Nurse’ and other titles, as well as those nurse professionals with a long-serving track record of experience and additional education in their field of expertise.

The challenges most often identified in the SN profession are the fragmentation of training and education, and a lack of recognition by position and title [2]. The literature shows that SNs have difficulties maintaining their role, regardless of educational preparation, role definition, and set competencies [5]. For some time now the term SN has been associated with the name of the job, not with a level of practice [14]. Such a complex framework complicates the mobility of SN nurses and the provision of quality care. This situation does not contribute to a sustainable health system—both in quality and economics—and above all it is not functional in terms of mobility [15].

The European Specialist Nurses Organization (ESNO) has begun the long-term process towards harmonization of SN education and recognition [16]. One of the ESNO’s goals is to gain recognition for the SN title in a European context, not only for reasons of mobility but also to address the advanced quality of care and patient safety [17]. Although the European Network for Nurses’ Organizations has developed a framework for specialist nursing education, to harmonize post-basic nursing education and to facilitate the free movement of SNs [18], in the recent directive on the recognition of professional qualifications we can observe that only general care nurses are represented, with no recognition of the important role of the SN [19]. This is an important gap, as SN roles have been established for many decades now and they play a significant role in healthcare delivery and outcomes. There is plenty of evidence available to show that SNs deliver effective outcomes and provide added value to healthcare [5].

This article does not only attempt to highlight the lack of recognition of SNs in the EU, but also to give meaning to this professional culture, which integrates expertise with advanced practices for the benefit of the quality of patient care. During the COVID-19 pandemic we have witnessed the problem of skills management arising in parallel with the shortage of equipment and medicines. The state of health of patients during the pandemic required the deployment of numerous wards to receive patients in respiratory distress. Mobilizing evidence on SNs in order to contextualize it with the managerial constraints encountered during the pandemic can be crucial for government decision-making on the need for SNs in the future. It is necessary to highlight the SN skills contributing to the management of care in the context of the massive patient flows in European healthcare institutions. Purpose of this study is to identify the interfaces of the specialist nurse profession across the EU and explore the standards of education, autonomy, and professional suggestions related to the identification of working conditions. ESNO gathers 15 different federations of nurses specialists who acts in all EU countries and for the purpose of the study we used actual network.

## 2. Materials and Methods

### 2.1. Design

A mixed method study design was used in a double-survey in two steps: we carried out a first quantitative study for the purpose of data collection of the countries’ and respondents’ characteristics, educational level, autonomy and responsibility, while qualitative methodology was used for analysing the open-ended questions as part of the second survey.

### 2.2. Sample

**Figure 1:**
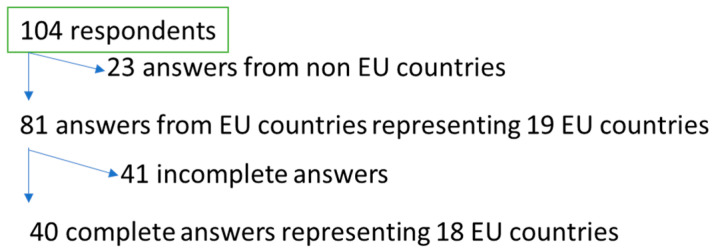
Flowchart of the SN organizations’ answers.

The data were collected in two parts, an initial quantitative exploration with a collection of responses from the leaders of the organizations and the following qualitative part sent directly to the organizations’ individual members. In the exploratory survey, we received 104 answers from SN organization respondents. Of these, 23 were removed because they were not from EU countries. The completeness of the answers was a second criterion; 41 answers were missing too many elements for analysis. The result was 40 usable answers completed from 18 different EU countries (Figure 1). Answers from following countries were included in study: Austria, Belgium, France, Croatia, Cyprus, Netherland, Finland, Greece, Hungary, Poland, Malta, Ireland, Norway, Portugal, Slovenia, Spain, Sweden and Denmark.

The flowchart below shows the steps to the final 40 eligible answers.

A total of 498 nurses participated in the second part of the survey. The majority of this sample were female nurses (375—75.3%) and the most frequent age reported was 35–44 years (151—30.3%).

### 2.3. Data Collection

The pilot study as exploratory phase was conducted by a SurveyMonkey^®^ online questionnaire sent to European SN organizations between 15 May and 15 June 2019. Many of the ESNO’s members have their own competence profiles, training frameworks, education routes, and even accreditation systems, but although there are a lot of overlaps, they are not related or unified. These educational and professional variables need evidence from in-depth studies to set up a harmonization and recognition strategy for SNs.

The main part of the study was also conducted by Survey Monkey^®^ over a period of 2 weeks between 2 October and 15 October 2019. To avoid the semantic constraints encountered in the first questionnaire, we opted to give more attention to the freedom of speech through open-ended questions. This approach was the right choice to take into account the cultural differences in terms of education and profession and so we collected a rich variety of interesting answers to submit to a statistical analysis.

### 2.4. Research Instruments

The quantitative study using close-ended questions was conducted by a SurveyMonkey^®^ online questionnaire sent to European SN organizations. The survey was based on the ECTS (European Credit Transfer System) users’ guide of the European Higher Education Area [20], completed with the recommendations for the European Qualifications Framework [21]. In the questions about educational programmes, we observed semantic misunderstanding because of national diversity, particularly in the answers about the ECTS, and the descriptors defining levels in the European Qualifications Framework were too heterogeneous and incoherent. The feedback was therefore not eligible for statistical analysis. A decision was made to design a more open and individually addressed questionnaire with open-ended questions integrating three dimensions for investigation: personal, educational, and professional profiles.

### 2.5. Data Analysis

Thematic analysis was used as a method for identifying, analysing, and reporting patterns (themes) within data. It is a widely used qualitative analytical method, mostly due to its flexibility. Since it is essentially independent of theory and epistemology, it can be applied across a range of theoretical and epistemological approaches [22]. Here it was applied in search of themes or topics of interest spontaneously mentioned by the SNs taking part in this research. The answers provided by the SNs, in particular to open-ended questions, made the data set used for our analysis. Each answer (from each individual participant) was one data item, and it could be coded as one or more extracts (one answer could have more than one code). In thematic analysis, the researcher has an active role and their judgement is necessary to determine what a theme is. According to Braun and Clarke, a theme captures something important about the data in relation to the research question, and represents some level of patterned response or meaning within the data set [22]. We aimed to preserve the richness of the data, because this appears to be an under-researched area and the findings would be further narrowed in expert validation. This means we identified as many extracts (codes) as possible, and later grouped them together into sub-themes and then major themes (a bottom-up or data-driven analysis).

## 3. Results

The results are structured into four categories. The first category is a presentation of the countries’ and respondents’ characteristics; in the second, the educational level is presented; autonomy and responsibility are in the third category; and the fourth and final category describes an analysis of the SNs’ suggestions related to the identification of conditions.

### 3.1. Demographic Data

#### 3.1.1. Countries’ and Respondents’ Characteristics

A total of 498 nurses with an average experience of 13 years participated in this research. The most represented were Malta (65—13.2%), Denmark (59—11.9%), France (56—11.3%), and the UK (United Kingdom) (48—9.7%). A quantitative analysis was used for the questions about educational level, autonomy and responsibility. The qualitative analysis was based on the free comments and suggestions.

#### 3.1.2. Educational Level

Most participants had completed either a 1st degree cycle (34.6%) or a 2nd degree cycle: Master’s 120 ECTS (35.6%). Almost 6% had completed doctoral studies. This diversity in the level of education has also been mentioned in other studies [2]. A total of 86 participants chose ‘other’ because they did not fit into the options that were offered to them or they needed to explain further. A majority (57.2%) followed a specific educational programme to become a SN.

#### 3.1.3. Autonomy and Responsibility

SNs are acquiring more professional autonomy (81.9%). The level of responsibility is mostly identified in order of task completion and behaviour in solving problems according to the circumstances (23.1%), managing complexity and professional development (19.5%), and also self-management within guidelines, supervision, evaluation and improvement of work (17.5%).

#### 3.1.4. Suggestions for the SN Profession

As the first step, reading through the items, we developed a coding list, and all the answers were coded. Only items with the same meaning were coded under the same code. All items considered unique, or bringing some new information, were coded under a different code so that we did not lose relevant data (hence, some items were grouped more than once if covering two or three themes in one answer).

### 3.2. Qualitative Research Results

**Figure 2:**
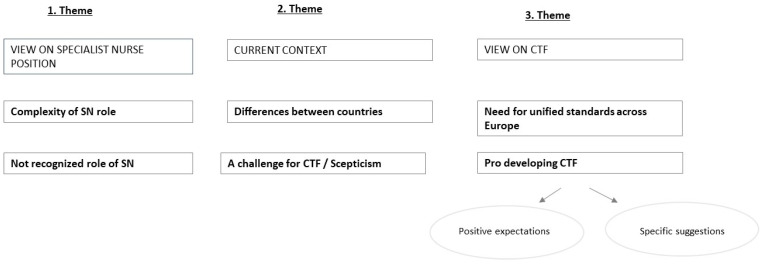
Summary of the findings.

In going through the items and the process of coding all individual answers, several topics could be extracted from the items (sub-themes), which were eventually grouped into several main themes, as presented in Figure 2.

We found that in answering the general question “Please share with us your suggestions about the SN profession in Europe”, the participants wrote about three main themes. They shared their view on the current position of SNs, their perception of current European conditions, and their view on a CTF (Common Training Framework). Each of these main themes contains two important sub-themes, and one sub-theme (the 3rd theme) further covers two sets of topics which were classified in separate sets of information for better clarity.

**Theme 1: View on the SN’s role**

Specialist Nurses from this sample shared their concerns about their current role and how it is perceived and recognized. They pointed out the complexity and importance of their role: “…*the Specialist role has both clinical and organizational accountabilities. Specialist Nurses should possess advanced clinical and decision-making skills and the ability to perform and implement Continuous Quality Initiatives, a performance improvement process, education, research and publication”. They feel under-appreciated by the system (legal, management, payroll, etc): “When professional skills evolve there could be a framework to recognize and pay for them. You get a diploma, and you try to do your best but there is no money for the time invested in the development”.*

*Subtheme 1.1: Complexity of SNs’ role*

The complexity of the SNs’ role is present in many aspects of the profession. The professional title may be an indispensable requirement for professional practice, “The Specialist Nurse title can be utilized for specialized practice such as Tissue Viability Nurse. For that title, postgraduate study is required, as the specialist role has both clinical and organizational accountabilities*”. The high professional level is also emphasized, making it possible to orient medical skills according to the healthcare needs at the time. “The most important thing is the high level of care that the specialist nurse profession can deliver. We do much of the work that an anaesthetist does so they can do more of the critical patient care”.*

*Subtheme 1.2: Not recognized role of SNs*

Sometimes the absence of a professional body makes it impossible to recognize the role of the nurse. “Unfortunately, although I have a public health nursing role, my role falls under public health consultants (who are doctors) who can limit the relevance and applicability of my work to the nursing context”. The CTF is proposed to obtain recognition from employers. “It will work well if we can be recognized, and have a CTF specific to the roles and the employers to support the programme”. A Master’s level with a well-defined framework are two elements to be taken into consideration for recognition. “I think that there must be a Master’s degree recognition of the SN; with an appropriate framework there could be a large span of action that could be taken by SNs”. Recognition requires autonomy, which is constrained by management. “We need more empowerment and flexibility to do what we are trusted with. More often than not it is the management that hinders my progression”. Even a regulatory framework does not guarantee recognition by employers. “In Italy the laws provide for SNs but is not recognized by companies, the system is very complex. I think that we need postgraduate courses like doctors”. A legal and official framework is essential for advanced practice. “We need to be covered officially and legally to perform our specialized advanced practices for the benefit of our patients”.; “Better salaries for the responsibilities. We are underpaid in France”.

**Theme 2: Current Context**

There is an important need to acquire expertise through the exchange of knowledge, know-how and interpersonal skills based on a common professional language. Nurses believe it is time to start unifying standards of education and job demands, so that SNs would have a more aligned position as healthcare professionals, regardless of which country they practice in: *“It would be good if specialist nurses became a reality in the whole of Europe, not only in some countries, with recognition both economically and by responsibility”. Specific suggestions the SNs gave covered a wide range of ideas, from “better defining a SN role” so that it is not left for interpretation to local authorities, to “more acknowledgement” given to some areas of specialization (anaesthesia, children, public health, etc.). The formalization of harmonized education for specialist nurses would be an asset for achieving mobility between countries and alleviating acute human resource shortages. This would allow managers to quickly find a balance of human resources to ensure continuity of care.*

*Subtheme 2.1: Differences between countries*

In some countries, the specialist nurse does not yet exist or must be developed, “Not every country has SNs”. There is great variability in education programmes and levels of responsibility. *“I know about nurse anaesthetists, and I do know that many European countries don’t even have them”. Even nationally, some SN qualifications are not well-defined by some countries. “The SN profession (Wound Care) is not well-defined officially in Belgium”. Disparity and lack of harmonization are mentioned in Europe, but the EU could help change that situation for the better. “European countries are not aligned, and some have advantages”. “My country has recently started a new classification system linked to our salaries. This system is not encouraging new professionals to become SNs”. Professional identity leaves room for specificities and must be respected by those in charge.*

*Subtheme 2.2: A challenge for CTF*

The CTF is a challenge in facing the different SN competences and its implementation. “*It’s challenging to develop a CTF with all the different levels of education and different autonomies”.* Cultural differences and politics hinder mobility projects in Europe. *“I am a fluent French speaker but I think that working in Europe would be too big a cultural step”. “I would like to network with my European counterparts, but I fear that they will marginalize the UK post-Brexit”. The ultimate managerial goal is to organize the work to ensure quality of care. For this reason, standardization and harmonization are essential to achieve evidence-based nursing.*

**Theme 3: View on the CTF**

The participants who support the idea of CTF believe that this is a good way to ensure the SN role and the need to be standardized and recognized across Europe—these are the main positive expectations. *“A very good initiative to benefit the patient, the nurse, and the profession”; “It is needed in every country”. Nurses believe it is time to start unifying standards of education and job demands, which would mean SNs would have a more aligned position as healthcare professionals, regardless of which country they practise in: “We need to have the sector stabilized with practice nurses all over Europe having the same work conditions and basic duties”. Europe’s macro-environment should allow for a levelling-out of the quality-of-care provision by SNs, supervised by healthcare management.*

*Subtheme 3.1: Need for unified standards across Europe*

The need for practice guidelines on regulation is a driving force for harmonizing education and research programmes. “Develop, together with specialist societies, uniform nursing training and study concepts that are recognized throughout Europe. Develop the nursing profession further together”. One goal is a similarity of practice and competence for all the SNs in Europe. “Equal curricula and accreditation between countries—standards”; “It’s time for equal international standards”; “Nomenclature needs to be standardized as a core topic”. Autonomy and responsibility acquired during training are professional qualities proper to specialized nurses by facilitating the organization of care.

*Subtheme 3.2: Pro developing CTF*

Positive expectations

The CTF is expected to have a positive impact on autonomy. *“This will increase our autonomy in the clinical field”.* The implementation of a CTF by speciality with a minimum educational level is an approach of excellence that creates a reference that could be exported beyond European borders. *“A structured approach is needed and definitely at a comprehensive Master’s degree level, in order to start out with well-developed research and thinking skills”.* Harmonization of education and requirements contributes to recognition and mobility, and improves the quality of care. *“Getting the same education and requirements would make it easier to get employment in other countries and to be able to evaluate work better”.* The recognition of SN professional experience based on formalized references is a managerial driver to integrate professionals trained elsewhere in one’s organization.

Specific suggestions

The role of the SN in European healthcare systems needs to be better defined. “SNs could be employed in a home care setting to prevent hospitalization and to improve quality of life”. Recognition could be achieved not only through the acquisition of knowledge, but also through experience. “Taking into account not only theoretical skills but also extensive experience and education throughout the years of employment”. A minimum of education level with or without professional experience is required”. A 2-year Master’s level with broad and specific knowledge is needed”. Continuing professional development with the possibility of in situ training is imperative and the acquired level of competency needs to be assessed. Specialized courses and face-to-face and online exchanges are suggested for the acquisition of knowledge: “More specialized courses”; “Meetings with nurses within the same specialist field”; “Obtaining information online”.

## 4. Discussion

In the search for excellence in healthcare through better quality and safety, Europe has put in place a policy to promote exchanges between countries for these health professionals and patients. EU directives go in this direction and provide a regulatory framework, as well as references for harmonization of qualifications and education. Knowing that the professional culture is already at a significant level of maturity, it is hard to believe in the lack of mutual recognition. So, Sondra Koff writes in her book about nursing in the EU: “The world of nursing specialization in the EU lacks uniformity. It is almost frenetic to temper the heterogeneity in the interests of quality practice and mobility is a formidable challenge” [23]. The principle of cross-border healthcare is unquestionably a universal goal for the SN profession. The decision-making autonomy of the Member States and the shortage of professionals are unfortunately cited among the barriers to achieving this objective. There are three EU directives that are linked to SN mobility: the recognition of professional qualifications and regulation [19,24]; the application of patients’ rights in cross-border healthcare [25] and the proportionality test [26], the most recent one. Looking for SN recognition, we need to analyse a CTF, which ‘means a common set of minimum knowledge, skills and competences necessary for the pursuit of a specific profession’ [16]. This approach “enables more professionals to move across Member States” (Ibid.). At a strategic level, it is important to take a stand for either a generic approach involving all specialist nurses, a cluster of specialist nurses, or a more specific group.

This is why a close look at SN practice is important. There is a need for specialized professionals not only in highly technical specialties, but also in chronic diseases such as TBC. Collin et al. found in his study that the “majority of countries identified the need for specialist training for nurses in tuberculosis patient care (56.7%)” [27]. Authors of a recent study concluded that “Advanced knowledge is a clear source of power for SNs in this context and in a facilitative work environment, the impact of this knowledge could become much more tangible than what their current experience shows” [28]. Other positive findings are in a paper by Coen and Curry: “The comprehensive and dynamic nature of the CNS’ (clinical nurse specialist) practice allows application of multiple roles across various care settings [29]. The CNS positively impacts outcomes, quality, and cost by implementing a unique set of skills and clinical expertise to improve not only direct patient care but also nursing practice and healthcare systems”. Begley et al. had the same idea and said that “ANPs (Advanced nurse practitioners) are considerable, including a higher level of patient/client care, increased leadership and greater research output” [3].

However, some authors underline several difficulties: “…advanced practice nurses struggle to negotiate and clarify scopes of practice while general practitioners have trouble justifying the costs associated with advanced practice nursing roles” [30]. Mayo concludes that the clinical nurse specialist “has a unique role to play in contributing to high quality patient care and system level change across multiple healthcare settings” [31]. The International Council of Nurses defines the standard of practice as “the range of roles, functions, responsibilities and activities, which a registered/licensed professional is educated for, competent in, and is authorized to perform” [32].

We are getting closer to a universal professional language and a more pragmatic level. We found the benefit of these standards in the literature. Some authors have gone further and found that this approach is “beneficial to patients: better adherence, reduced waiting times, improved effectiveness of resource management, quality of care comparable with that of physician experts” [17]. However, there is some criticism about EU policy. Ranchal et al. said that “the EU has failed to standardize nursing outside essential criteria for general nurse and midwifery training. Definitions of ‘basic’ nursing exist but are not sufficient, for nursing cannot solely be defined by its core function; it must also be defined by its cutting edge, its direction and its current” [15]. In addition, they stated that the lack of standardization is not the most important issue, but that professional mobility defines the very purpose of the EU.

## 5. Conclusions

All this progress led us to identify, step by step, using quantitative and qualitative data, the priorities of a strategy in view of the harmonization and mobility of specialized nurses. The professionals themselves can identify the conditions in the work field with real awareness and consciousness of their input. For professionals, these elements are also the grounds for claiming greater recognition, which need to be endorsed by healthcare managers. A striving for homogeneity in the interpretation of the SN role and competencies is needed to achieve better quality of care provision and facilitate their mobility across the EU.

The lack of recognition identified in this study should encourage nurse managers to consider SN roles with the aim of capitalizing on the advanced care and expertise that SNs provide. These results are an opportunity to improve the SN profession with an ultimate impact on management practices of streamlined, cost-effective clinical services. Findings of this study will represent baseline for further research and development of specialist nurses in Europe.

## Data Availability

The data and the questionnaires of the study are available upon request from the corresponding author.
